# Broadband photon pair generation in green fluorescent proteins through spontaneous four-wave mixing

**DOI:** 10.1038/srep24344

**Published:** 2016-04-14

**Authors:** Siyuan Shi, Abu Thomas, Neil V. Corzo, Prem Kumar, Yuping Huang, Kim Fook Lee

**Affiliations:** 1 Center for Photonic Communication and Computing, Department of Electrical Engineering and Computer Science, Northwestern University, 2145 Sheridan Road, Evanston, IL 60208-3112 USA; 2 Department of Physics and Engineering Physics, Stevens Institute of Technology, Hoboken, NJ 07030 USA

## Abstract

Recent studies in quantum biology suggest that quantum mechanics help us to explore quantum processes in biological system. Here, we demonstrate generation of photon pairs through spontaneous four-wave mixing process in naturally occurring fluorescent proteins. We develop a general empirical method for analyzing the relative strength of nonlinear optical interaction processes in five different organic fluorophores. Our results indicate that the generation of photon pairs in green fluorescent proteins is subject to less background noises than in other fluorophores, leading to a coincidence-to-accidental ratio ~145. As such proteins can be genetically engineered and fused to many biological cells, our experiment enables a new platform for quantum information processing in a biological environment such as biomimetic quantum networks and quantum sensors.

Recent progresses in quantum biology^1^, such as radial-pair magnetoreception^2^,^3^, quantum coherent excitation energy transfer in photosynthesis^4^,^5^,^6^,^7^, environment assisted quantum transport^8^,^9^, and quantum entanglement in photosynthetic complexes^10^,^11^, have signified quantum mechanics as a prospective tool for a multitude of new applications in biological systems. A new platform for quantum information processing in a biological environment can be developed for the realization of practical biomimetic quantum networks^12^ and quantum sensors^13^ in the future.

Among other biological materials, the protective beta-barrel structure of the green fluorescent protein (GFP) plays an important role in increasing quantum efficiency of absorption-emission process from 80%^14^ to 93%^15^ and developing the GFP laser in solid state form^16^. A single-cell biological laser based on GFP has been demonstrated^17^ because of its feasibility in expressing and fusing to biological cells. In enhanced green fluorescent protein (eGFP), the fluorophore is located in the interior of the beta-sheet barrel conformation^16^. The eGFP consists of three amino acid residues (Thr65, Tyr66, Gly67) that are responsible for the green fluorescence. The beta-barrel protects the fluorophore from the environmental interference caused by adjacent fluorescent proteins. Yet many potential photon-mediated nonlinear quantum processes and their quantum states of light emitted from the fluorophores of the fluorescent proteins remain to be explored. Therefore, it is important to develop a universal empirical method based on spectral and polarization filtering for analyzing relative strength of nonlinear optical interaction processes such as four-wave mixing, two-photon excitation and Raman scattering process in fluorescent proteins.

In this work, we explore quantum nonlinear optics in such proteins. Specifically, we generate broadband (~20 nm) photon pairs through spontaneous four-wave mixing process in eGFP. We study the role of beta-barrel structure in fluorescent proteins by analyzing and comparing the relative strength of spontaneous four-wave mixing, two-photon excitation and Raman scattering process in other organic fluorophores without such structure. High-quality photon pairs are generated in eGFP exhibiting a coincidence to accidental ratio (CAR) ~145, the highest obtainable value among other fluorophores tested. This indicates that the beta-barrel structure in fluorescent protein can suppress environmental interference between the fluorophores of adjacent fluorescent proteins, giving rise to a short quantum coherence time in photon-pair emission, which is provided by a 200 fs excitation pump pulse. Since the eGFP can be genetically engineered and expressed to biological cells^18^,^19^,^20^,^21^,^22^,^23^,^24^, our demonstration of photon-pair generation in fluorescent proteins could be a step toward developing a new platform for quantum information processing in a biological environment.

## Results

### Conversion Efficiency of four-wave mixing process

**Figure 1:**
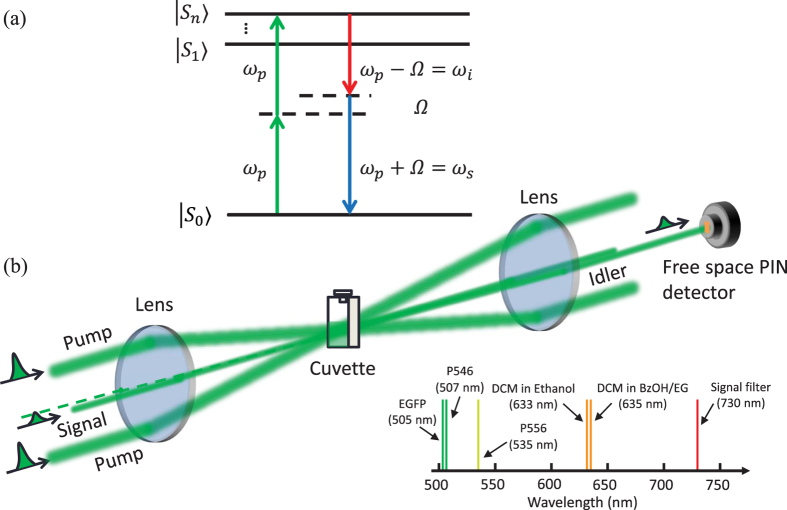
(**a**) The energy diagram of the eGFP. Four-wave mixing process on the excited vibronic states (*S*_*n*_, *S*_1_) and the ground state (*S*_0_) of the eGFP. (**b**) A pump-probe configuration setup for measuring the conversion efficiency of four-wave mixing process. Inset: the locations of fluorescence peak of each sample and the signal filter. P556(pyrromethene 556), P546(pyrromethene 546).

In four-wave mixing process, two pump photons at frequency *ω*_*p*_ scatter through the *χ*^3^–nonlinearity of the fluorophore and create signal-idler photon pairs at frequencies *ω*_*s*_ and *ω*_*i*_, such that their energy (2*ω*_*p*_ = *ω*_*s*_ + *ω*_*i*_) is conserved. The virtual energy level of the four-wave mixing process is shown in Fig. 1(a). We prepare five samples; (a) eGFP with a molar concentration of 25.5 *μ*M in phosphate buffered saline (Biovision^®^), (b) DCM (4-(dicyanomethylene)-2-methyl-6-(p-dimethylaminostyryl)-4H-pyran) with a molar concentration of 0.99 mM in ethanol (Exciton^®^), (c) DCM with a molar concentration of 1.5 mM in mixed solvent of benzyl alcohol/ethylene glycol (BzOH/EG) with a ratio of 2/3 (Exciton^®^), (d) pyrromethene 556 with a molar concentration of 4.3 mM in EG (Exciton^®^), and (e) pyrromethene 546 with a molar concentration of 250 *μ*M in methanol (Exciton^®^). The solvents (phosphate buffered saline, ethanol, BzOH/EG, EG and methanol) do not contribute to FWM efficiency. The concentration of each sample is prepared according to the instructions provided by the vendors. For the eGFP, the beta-barrel prevents close contact between fluorophores, and hence allows a molar concentration as high as 1 mM before the protection becomes ineffective^16^. We prepare the eGFP with a molar concentration well below 1 mM for generating photon pairs through spontaneous four-wave mixing process. Among the 5 different samples, the GFP is the only sample that has the protective beta-barrel structure. There are two main reasons for choosing these samples. First, we want to show that the GFP can provide photon pair with a high CAR because of the structure. Second, we want to develop an empirical method based on polarization and spectral filtering for systematically studying the relative strength of nonlinear optical interaction processes in 5 different organic fluorophores.

**Figure 2:**
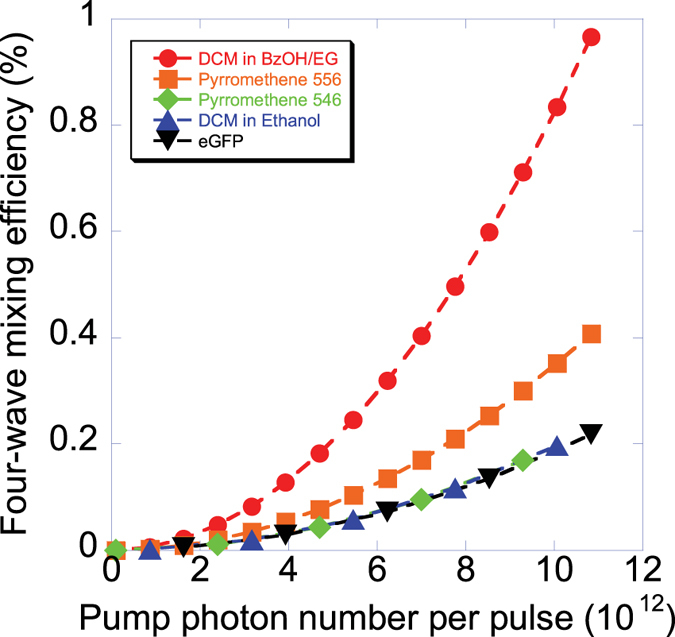
The conversion efficiency of stimulated four-wave mixing process in five organic fluorophores.

Our first demonstration is to measure the conversion efficiency of four-wave mixing process for each sample by using the stimulated four-wave mixing process in a pump-probe configuration (see Fig. 1(b)). The efficiency of the four-wave mixing process is quadratically dependent on pump power as shown in Fig. 2. Our results confirm that four-wave mixing process occurs in all these samples. We are the first to measure the four-wave mixing conversion efficiency on these samples in the degenerate forward four-wave mixing scheme at 785 nm. Our efficiency of these samples are comparable to the efficiency of dyes in thin films^25^,^26^,^27^,^28^ range from 0.01% to 1%. We also observe that the DCM in the BzOH/EG and the pyrromethene 556 have higher efficiency than the DCM in ethanol, pyrromethene 546 and eGFP because of the concentration and solvents of these samples. However, later, we show that their efficiency is not directly implying the high CAR of photon pairs generated from spontaneous four-wave mixing process because of noise photons generated from two-photon excitation and Raman scattering.

### Photon-pair generation and noise photons

**Figure 3:**
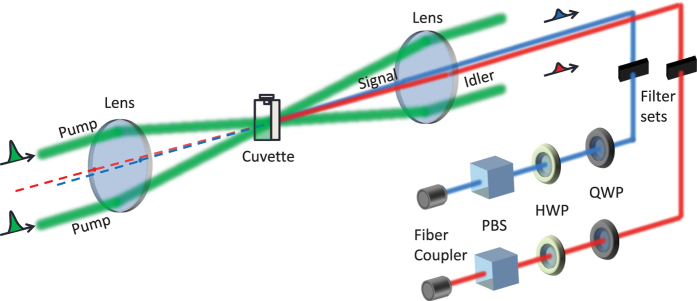
Experimental setup for photon-pair generation based on a forward spontaneous four-wave mixing process.

The experimental setup is shown in Fig. 3, see the details in Methods. The two pump beams are obtained from a mode-locked regenerative amplifier (Coherent Inc., RegA-9000 seeded by Mira-900 and pumped by Verdi-10) with repetition rate of 40 kHz and pulse duration of 200 fs at the center wavelength of 785 nm (full width at half maximum (FWHM) bandwidth of 10 nm). The total average power of two pump beams for the experiment can be varied from 2 to 110 mW. The signal and idler are generated at 730 nm and 849 nm, respectively. We study the nonlinear quantum processes of five different samples, where their fluorescence peaks are located at the wavelengths shorter than the center wavelength of the signal filter at 730 nm as shown in the inset of Fig. 1(b). With sufficient high peak pump power, the fluorescence of two-photon excitation can scatter into the signal and idler channels. Recently, a frequency-upconverted stimulated emission by five-photon absorption has been observed in fluorophore^29^. The stimulated emission may occur in our experiment but its spectrum is a factor of 3 narrower than the fluorescence spectrum^29^, so the stimulated emission can be more effectively suppressed by using the spectral filtering as shown in the inset of Fig. 1(b). In addition to the fluorescence and stimulated emission generated from two-photon excitation, Raman scattering processes can occur. A single pump photon scatters inelastically by annihilating (anti-Stoke process) and creating (Stoke process) a vibrational phonon in fluorescent proteins. As a consequence, the Raman anti-Stokes and Stokes photons will appear in the signal and idler channels, respectively. The contribution of Raman photons can be distinguished by observing the linear power dependence on the signal and idler^30^.

We perform photon counting on the signal and idler generated from the samples. The number of the recorded signal and idler photons are given as 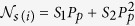, where *P*_*p*_ is the pump photon per pulse. The *S*_1_ and *S*_2_ are the linear and quadratic power dependence scattering coefficients, respectively. See the Methods for the physical parameters of *S*_1_ and *S*_2_. The *S*_1_ is corresponding to the total strength of spontaneous (SpRS) and stimulated Raman scattering (SRS) processes, i.e., 
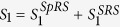
. The *S*_2_ is corresponding to the total strength of the spontaneous four-wave mixing (FWM) process, the fluorescence (FL) and stimulated emission (SE) components of two-photon excitation, i.e., 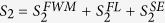. In experiment, we measure *S*_1_, *S*_2_, and the CAR (analogous to the signal-to-noise ratio)^31^,^32^ with and without polarization filtering for carefully characterizing the photon pairs generated in fluorescent proteins. In our detection system, a coincidence count is recorded when both single photon counting modules (SPCMs) detect a photon in the same gated time interval, while an accidental coincidence count is recorded when both SPCMs detect a photon in the adjacent gated time intervals. After we subtract the detector dark counts and background photons, we obtain the CAR value. The background photons are measured by blocking the pump beams from the laser. For the clarity of our CAR value, for example, the CAR of 20 means that 1 out of 20 coincidences is accidentals. The CAR > 1 indicates that there is quantum correlation between the photons in the signal and idler channels. Methods to characterize the purity of photon pairs^33^,^34^ and to extract Raman scattering photon from the CAR measurement^35^,^36^ have been demonstrated.

The *S*_1_ and *S*_2_ coefficients have been used to study the contribution of Raman scattering and four-wave mixing, respectively, in a dispersion-shifted fiber^30^,^37^. In the present work, we include polarization filtering in the *S*_1_ and *S*_2_. We define a function 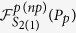 for the 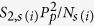 and *S*_1,*s*(*i*)_*P*_*p*_/*N*_*s*(*i*)_ (*s*-signal and *i*-idler), where *p*(*np*) denotes the experiment with (without) using polarization filtering on the signal and idler channels. See the Methods for the definition of 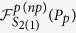. We include polarization filtering in these functions because of the polarization properties of photons generated through the four-wave mixing and other nonlinear noise sources. See the Methods for the polarization properties of photons in signal and idler channel. We use the function 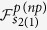 and the criterion of the CAR ≥ 1 for analyzing relative strength of nonlinear optical interaction processes and identifying the dominant quantum nonlinear processes in organic fluorophore as shown in Table 1.

#### DCM samples

**Figure 4:**
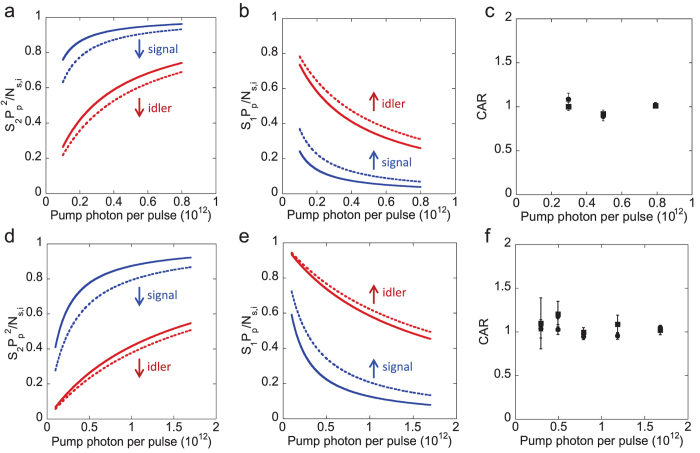
For the DCM in BzOH/EG sample, (**a**) 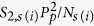, (**b**) *S*_1,*s*(*i*)_*P*_*p*_/*N*_*s*(*i*)_, without (solid line)/with (dotted line) polarization filtering (PF), and (**c**) the CAR. For the DCM in ethanol sample, (**d**) 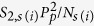, (**e**) *S*_1,*s*(*i*)_*P*_*p*_/*N*_*s*(*i*)_, without (solid line)/with (dotted line) polarization filtering (PF), and (**f**) the CAR. ↑ (increase), ↓ (decrease), the CAR (square) with PF, CAR (circle) without PF, raw CAR (diamond) with PF, and raw CAR (triangle) without PF. All CARs are overlapped to each others. Raw: without the subtraction of background photons and detector dark counts. After we subtract the detector dark counts and background photons, we obtain the CAR value.

When the DCM samples are excited by two-photon excitation process, the fluorescence peak of the DCM in BzOH/EG (Ethanol) is at 635 nm (633 nm) with a FWHM of 50 nm (80 nm), respectively. The recorded signal and idler are shown in the supplementary Fig. S1. We plot 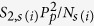 for the DCM samples as shown in Fig. 4(a,d). The plots (↓) show that the quadratic component of the signal and idler is decreased by polarization filtering. Even though the polarization filtering reduces the number of fluorescence, stimulated emission and spontaneous Raman photons by half, our analysis (see Eq. (2)) indicates that the decrease of 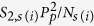 (Table 1; 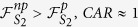) is due to the polarized Stokes and anti-Stokes generated by the stimulated Raman scattering, i.e., 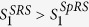. The stimulated Raman photon remains constant in *N*_*s*(*i*)_ with and without using the polarization filtering. Another interesting feature is the plot of *S*_1,*s*(*i*)_*P*_*p*_/*N*_*s*(*i*)_ for the DCM samples in Fig. 4(b,e). The plots (↑) show that the increase of the contribution/percentage of linear power component when the polarization filtering is used for the signal and idler. Since spontaneous Raman photon, the fluorescence and stimulated emission photons are not polarized, the increase (see the Methods, Eq. (3) and Table 1; 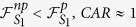) is mainly due to the fact that there is more reduction of the fluorescence and stimulated emission photons than the spontaneous Raman photon (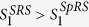, 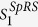 is weak) by the polarization filtering. We plot the CAR on the signal and idler photons generated from the DCM samples. Not surprisingly, we obtain the CAR ≈ 1 for the scenario of with and without using polarization filtering as shown in Fig. 4(c,f) for the DCM in BzOH/EG and Ethanol, respectively. The CAR of 1 indicates that the fluorescence, stimulated emission and stimulated Raman photons suppress the quantum correlation of signal and idler generated through spontaneous four-wave mixing process (see Table 1; 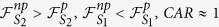). Even though the fluorescence and stimulated emission are quadratically dependent on power, the fluorescence contributes more noise photons than the stimulated emission at the signal channel because of the spectral filtering. Even though the DCM in BzOH/EG has high conversion efficiency in the stimulated four-wave mixing process (Fig. 2), but the DCM cannot provide high-quality photon pairs because the stimulated Raman scattering and two-photon excitation are the dominant nonlinear processes.

#### Pyrromethene 556

**Figure 5:**
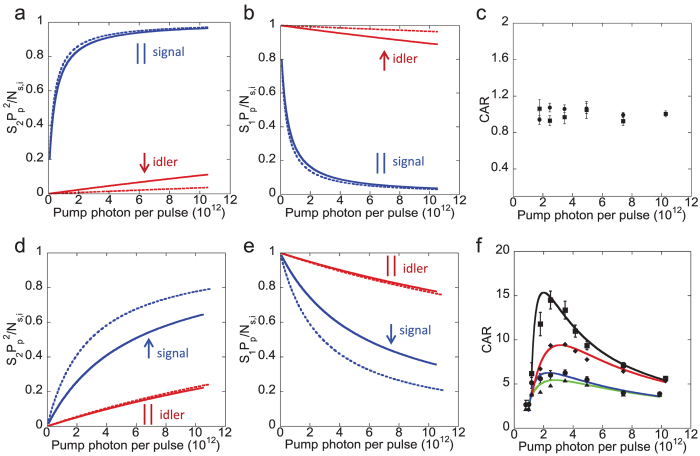
For the pyrromethene 556 sample, (**a**) 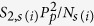, (**b**) *S*_1,*s*(*i*)_*P*_*p*_/*N*_*s*(*i*)_, without (solid line)/with (dotted line) polarization filtering (PF), and (**c**) CAR. For the pyrromethene 546 sample, (**d**) 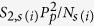, (**e**) *S*_1,*s*(*i*)_*P*_*p*_/*N*_*s*(*i*)_, without (solid line)/with (dotted line) polarization filtering (PF), and (**f**) CAR (The solid lines are theoretical fits^35^,^36^,^54^,^55^). ↑ (increase), ↓ (decrease), || (unchanged), the CAR (square) with PF, CAR (circle) without PF, raw CAR (diamond) with PF, and raw CAR (triangle) without PF. Raw: without the subtraction of background photons and detector dark counts.

For this sample, the fluorescence peak of two-photon excitation is at 535 nm with a FWHM of 50 nm. The recorded signal and idler are shown in the supplementary Fig. S2. We observe the CAR ≈ 1 as shown in Fig. 5(c). We plot 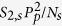 and *S*_1,*s*_*P*_*p*_/*N*_*s*_ for the signal as shown in Fig. 5(a,b). The plots show that there is no effect (indicated as ||) whether or not polarization filtering is applied. This indicates that the spontaneous Raman scattering, fluorescence and stimulated emission of two-photon excitation in the signal are the dominant nonlinear processes as shown in Table 1 (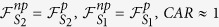). We then plot 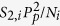 and *S*_1,*i*_*P*_*p*_/*N*_*i*_ for the idler as shown in Fig. 5(a,b). The plots show the similar behavior as previously discussed for the DCM samples (Table 1; 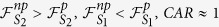). The criterion indicates that the dominant noise photons are fluorescence, stimulated emission, and stimulated Raman photons.

#### Pyrromethene 546

The fluorescence peak of two-photon excitation for the pyrromethene 546 sample is at 507 nm with a FWHM of 40 nm. The recorded signal and idler are shown in the supplementary Fig. S3. For the pyrromethene 546 sample, we observe the maximum CAR of 6 as shown in Fig. 5(f) for the scenario where the polarization filtering is not applied. The maximum CAR increases to 15 when we use the polarization filtering to reject the cross-polarized noise photons from spontaneous Raman scattering, the fluorescence and stimulated emission from two-photon excitation. We plot 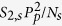 and *S*_1,*s*_*P*_*p*_/*N*_*s*_ for the signal as shown in Fig. 5(d,e). The plots show the opposite behavior as observed in the DCMs and pyrromethene 556. The increase of 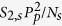 (↑) and the decrease of *S*_1,*s*_*P*_*p*_/*N*_*s*_ (↓) by using the polarization filtering in the signal are due to the reduction of spontaneous Raman photon, as shown in the Table 1 (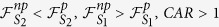). As for the idler, stimulated Raman scattering is the dominant noise source as shown in Table 1 (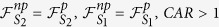). From here, we learn that the spontaneous and stimulated Raman scattering processes are present in the signal and idler channels, respectively. However, these processes in pyrromethene 546 sample are not as strong as in DCM samples and pyrromethene 556.

#### eGFP

**Figure 6:**
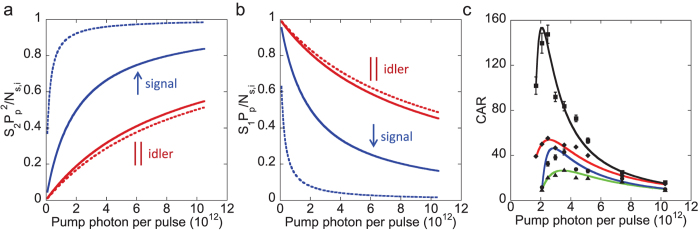
For the eGFP, (**a**) 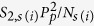, (**b**) *S*_1,*s*(*i*)_*P*_*p*_/*N*_*s*(*i*)_, without (solid line)/with (dotted line) polarization filtering (PF) and (**c**) CAR (The solid lines are theoretical fits^35^,^36^,^54^,^55^). ↑ (increase), ↓ (decrease), || (unchanged), the CAR (square) with PF, CAR (circle) without PF, raw CAR (diamond) with PF, and raw CAR (triangle) without PF. Raw: without the subtraction of background photons and detector dark counts.

The fluorescence peak of two-photon excitation for the eGFP sample is at 505 nm with a FWHM of 40 nm. The recorded signal and idler are shown in the supplementary Fig. S4. For the eGFP sample, we observe the maximum CAR of 45 without using polarization filtering. The spontaneous four-wave mixing process in eGFP is much stronger than in the pyrromethene 546. We observe the maximum CAR of 145 by using polarization filtering as shown in Fig. 6(c). This high CAR can provide two-photon interference with the visibility of 
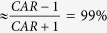
. We plot 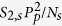 and *S*_1,*s*_*P*_*p*_/*N*_*s*_ for the signal as shown in Fig. 6(a,b). The increase of 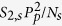 (↑) and the decrease of *S*_1,*s*_*P*_*p*_/*N*_*s*_ (↓) for the signal are more dramatic compared to the pyrromethene 546 sample. This indicates that the spontaneous Raman photon is the dominant noise source but much less than the signal photon generated through spontaneous four-wave mixing process as shown in Table 1 (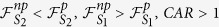). We plot 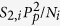 and *S*_1,*i*_*P*_*p*_/*N*_*i*_ for the idler in Fig. 6(a,b). The plots show no effect (indicated as ||) of using the polarization filtering. This indicates that the stimulated Raman scattering is the dominant noise source as observed by the idler in the pyrromethene 546 sample (Table 1; 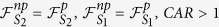). There is much stronger strength of 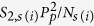 and lesser strength of *S*_1,*s*(*i*)_*P*_*p*_/*N*_*s*(*i*)_ observed in eGFP than the strengths observed in the pyrromethene 546 sample. Our analysis does not imply that the spontaneous four-wave mixing process in the eGFP is the strongest among other samples. We imply that the eGFP is the less noisy environment for the generation of photon pairs through spontaneous four-wave mixing process. We summarize the above observation for each sample as shown in Table 2.

## Discussion

For the eGFP and the pyrromethene 546 with polarization filtering, we obtain the maximum CAR values of 145 and 15, respectively. At the peak pump power around 2.5 × 10^6^ W, the photon production rate 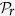 is around 7 × 10^−4^/pulse for the signal and 1.0 × 10^−3^/pulse for the idler, respectively. Among all organic fluorosphores tested, the eGFP emits highest-quality, broadband (20 nm) photon pairs, characterized by the quantum correlation (CAR ~145). This can be attributed to the fact that the fluorophore of the fluorescent protein, and thus its quantum nonlinearities, is protected by the beta-barrel, avoiding molecular aggregation which leads to fluorescence quenching, collision quenching, and fluorescence polarization between two adjacent fluorescent proteins. The two-photon excitation^38^ and Raman scattering can decohere the mechanism of photon-pair generation by changing the electrostatics environment of the fluorophore. As a consequence, noise photons are generated in all five samples. The beta-barrel in eGFP protects the electrostatic environment of the fluorophore from adjacent fluorophores and hence prevents the protein from environmental decoherence. Our results indicate that the generation of photon pairs in eGFP occurs in less noisy environment compared to other fluorophores. The CAR of 145 obtained in the eGFP is comparable to the results obtained in fibers^31^,^32^,^39^,^40^,^41^ and on chips^42^,^43^.

The empirical formula of 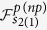 incorporated with the CAR measurement can provide a systematic study on the quantum origin of signal and idler generated in a complex biological system. The spectral and polarization filtering can extract out the information of many quantum processes taking place simultaneously in a complex organic system by means of selectively projecting out correlated photon pairs from a spectrally and polarization incoherent environment.

The photobleaching effects in fluorescent proteins have been observed in the two-photon excitation processes^44^,^45^. Surprisingly, we did not observe the photobleaching effect on the photon-pair generation in eGFP (peak irradiance: 50 GW/cm^2^ at CAR ≈ 145, repetition rate: 40 kHz) because the spontaneous four-wave mixing is related to the instantaneous response of the real part of *χ*^3^-nonlinearity. This motivates the use of photon pairs generated in the eGFP expressing cells for biosensing such as measuring the change of the refractive index of a cell. However, as we operate the pump pulse at 60 kHz with the average power of each pump about 75 mW, the four-wave mixing efficiency is decreased. The lifetime of the eGFP sample in our experiment is at least 6 months.

In our previous work in fibers^31^,^32^, we obtained CAR > 100 for the narrow-band (1 nm) photon pairs generated in optical fiber at 77 K. One may think that the broad bandpass filters can allow more noise photons at different modes in the photon-pair channels and decrease the CAR. On the contrary, we have the maximum CAR ~145 for the 20 nm-bandwidth photon pairs generated in eGFP. The noise photons are mainly attributed to the spontaneous and stimulated Raman scatterings, which can be mitigated by reducing the detunning of photons from the pump, and/or using polarizers to remove the cross-polarized Raman photons. Another option is to reduce the phonon occupation of vibrational Raman modes by cooling the eGFP to liquid nitrogen temperature^4^,^5^. The CAR of 145 reported here in eGFP can provide theoretical upper limits of the visibility of two-photon interference as 
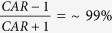
, that is after the subtraction of the detector dark counts and background photons. Our four-wave mixing scheme can be used to generate time-energy entanglement^46^,^47^ for quantum information processing. With such a high CAR source, we believe that the photon pair source can be benefit for some quantum communication applications^48^. As a high CAR can suggest a good visibility, the photon pair source can be tested for other quantum interference applications^49^,^50^.

## Methods

### Nonlinear Quantum Processes in GFP

The number of signal and idler photons generated can be expressed as^51^,^52^,^53^,


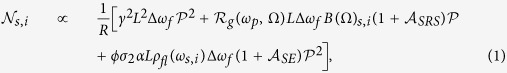


where *R* is the repetition rate of the pump with the peak power 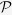, 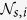 is the number of signal or idler photons per pulse. In Eq. (1), the first term is the spontaneous four-wave mixing process, where the *γ* is the nonlinear coupling coefficient, Δ*ω*_*f*_ is the filter bandwidth for the signal and idler, and *L* is the length of the sample. The second term is the spontaneous and stimulated Raman scattering process, where Raman gain 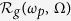
51, Ω = *ω*_*p*_ − *ω*_*i*_ = *ω*_*s*_ − *ω*_*p*_, *B*(Ω)_*s*,*i*_ is Bose phonon population factor for the signal and idler, and 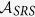 is the probability of the initial number of signal or idler being participated in the stimulated Raman scattering process. The third term is the fluorescence and stimulated emission components of the two-photon excitation process^52^, where *ϕ* is the fluorescence emission efficiency due to the two-photon excitation with the cross section of *σ*_2_, *α* is the density of molecule in the sample, *ρ*_*fl*_(*ω*_*s*,*i*_) is the normalized fluorescence spectrum lineshape at the signal and idler, and 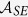 is the probability of having stimulated emission^53^.

### Polarization properties of photons in signal and idler channel

It is a fundamental challenge to distinguish the photon pairs from noise photons generated in the organic fluorophores. We can minimize the noise photons in the photon-pair channels by using spectral and polarization filtering. In our experiment, the signal and idler generated through the spontaneous four-wave mixing process are co-polarized with the pump, quadratically dependent on power, spectrally correlated by the relationship of 2*ω*_*p*_ = *ω*_*s*_ + *ω*_*i*_. On the contrary, the fluorescence photon emitted from two-photon excitation is randomly polarized, quadratically dependent on power, spectrally uncorrelated, and their spectrum is dictated by the transitions from the excited vibronic states (*S*_1_) to the ground vibronic states (*S*_0_). The polarization of the stimulated emission photon is not dependent on the polarization of the pump. The spontaneous Raman photon is also randomly polarized in average, linearly dependent on power, and spectrally uncorrelated. However, the stimulated Raman photon is co-polarized with the pump, linearly dependent on power and spectrally uncorrelated. Of these, we can characterize the signal and idler generated in the organic fluorophores as follow; (i) photon counting on *N*_*s*_ and *N*_*i*_ with and without using polarization filtering, and (ii) performing the CAR measurement.

### Empirical formula for relative strength of nonlinear quantum processes

The empirical formula for the function 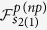 is given by,


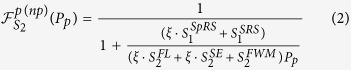



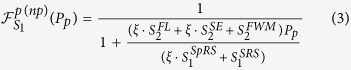


where the *ξ* = 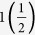 is for *np*(*p*) and 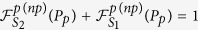. The factor of 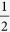 is due to the polarization filtering for the unpolarized (in average) noise photons such as spontaneous Raman photon, the fluorescence, and stimulated emission photons of two-photon excitation.

There are 3 categories for the combinations of 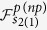; 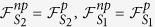; 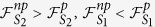; and 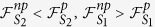. For each category, we characterize the nonlinear quantum processes based on the CAR ≈ 1z and >1 (see the Table 1).

### A pump-probe configuration

As shown in Fig. 1(b), the two pump beams and one probe (signal) beam are obtained from a mode-locked regenerative amplifier (Coherent Inc., RegA-9000 seeded by Mira-900 and pumped by Verdi-10) with repetition rate of 40 kHz and pulse duration of 200 fs at the center wavelength of 785 nm. The average power of three beams can be controlled by a half wave plate (HWP) and a polarizing beam splitter. The three spatially separated and co-polarized beams are focused inside a sample by using a lens of *f*_1_ = 50 cm. The beam waist for each beam is about 40 *μm* corresponding to confocal parameter (twice the Rayleigh range) of 13.9 mm. The two pump beams propagate in a vertical plane while the probe signal and the generated idler beams propagate in a horizontal plane. Each sample is kept in a 5-mm long quartz cuvette. After the sample, we use a lens of *f*_2_ = 2*f*_1_ = 100 cm to collect and collimate the generated idler beam. The idler beam is generated at the wavelength of 785 nm and located at the opposite position of the the probe signal beam.

### Experimental setup for photon-pair generation

Figure 3 is used for exploring the forward spontaneous four-wave mixing process and other nonlinear quantum processes that may occur in fluorescent proteins. In contrast with the previous experiment, we block the probe signal beam in front of the sample and only use two pump beams to generate signal and idler photons, which are non-degenerate. In the forward spontaneous four-wave mixing process, two pump photons are annihilated to create signal and idler photons while conserving their energy (2*ω*_*p*_ = *ω*_*s*_ + *ω*_*i*_) and momentum (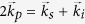), where the pumps, signal and idler are not collinear propagating beams. The phase mismatching of the four-wave mixing process is given by 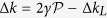, where Δ*k*_*L*_ is due to the linear dispersion and the *γ* is nonlinear coupling coefficient. The 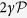 is nonlinear contribution from the pump power (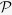)^51^ such as self-phase modulation and cross-phase modulation. A delay line (not shown in Fig. 3) is used to compensate the path difference between the two pump beams so they arrive simultaneously at the focal spot. After the sample, we use a notch filter with 3-dB bandwidth of 33 nm at the center wavelength of 785 nm for blocking the scattered pump photons in the signal and idler directions. The use of a lens of *f*_2_ = 2*f*_1_ after the sample is to increase the spatial separation between the pumps, signal, and idler by a factor of two, so keeping the vast majority of the unblocked pump photons away from the signal and idler paths.

We select the signal and idler photons at the center wavelengths of *λ*_*s*_ = 730 nm and *λ*_*i*_ = 849 nm, respectively. These signal-idler wavelengths are far-detuned from the spectrum of the pump photons. The spectral isolation is obtained by using tunable bandpass filters (TBFs) with 3-dB bandwidth of 20 nm and single-pass transmission efficiency of 97%. The transmission wavelength of the filter can be tuned by changing the angle of incidence. We place two cascaded TBFs on a rotation stage in each signal and idler. The double-passing scheme with a retro-reflector provides an isolation >140 dB from the pump photons. We then use a free-space-fiber collimator to couple signal/idler photon to SPCM. We set up polarization measurement device which consists of a half-wave plate, a quarter-wave plate and a cube polarizing beam splitter, on each signal and idler. The combination of a half-wave plate and a quarter-wave plate is used to compensate the birefringence of the photon pairs on each optical component in the experiment. The SPCM is active for the time duration about 45 ns, which is much larger than the FWHM of the coincidence peak of the signal and idler photons. The dark count probability of the SPCM is about 10^−5^. The total detection efficiency for the signal (idler) is 27% (23%), respectively. We record photon counting for the signal and idler photons, and then use a correlator (CPDS, Nucrypt LLC) for measuring coincidences and accidentals. In our detection system, a coincidence count is recorded when both SPCMs detect a photon in the same gated time interval, while an accidental coincidence count is recorded when both SPCMs detect a photon in the adjacent gated time intervals.

## Additional Information

**How to cite this article**: Shi, S. *et al.* Broadband photon pair generation in green fluorescent proteins through spontaneous four-wave mixing. *Sci. Rep.* **6**, 24344; doi: 10.1038/srep24344 (2016).

## Supplementary Material

Supplementary Information

## Figures and Tables

**Table 1:** The use of 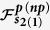 for characterizing the dominant nonlinear processes in organic fluorophore.

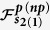	CAR	relative strength of nonlinear process	dominant process
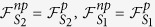	CAR ≈ 1	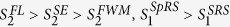	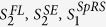
	CAR > 1	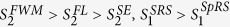	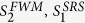
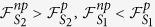	CAR ≈ 1	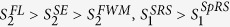	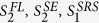
	CAR > 1	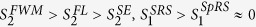	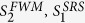
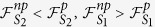	CAR ≈ 1	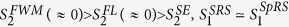	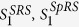
	CAR > 1	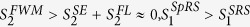	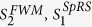

**Table 2:** A summary of the maximum CAR and dominant processes of 5 organic fluorophores.

Sample	MaximumCAR	Dominantprocess (signal)	Dominantprocess (idler)
DCM in BzOH/EG	1	FL, SE, SRS	FL, SE, SRS
DCM in ethanol	1	FL, SE, SRS	FL, SE, SRS
Pyrromethene 556	1	FL, SE, SpRS	FL, SE, SRS
Pyrromethene 546	15	FWM, SpRS	FWM, SRS
eGFP	145	FWM, SpRS	FWM, SRS
