# Supplementary material for: Broadband photon pair generation in green fluorescent proteins through spontaneous four-wave mixing
Source: Sci Rep. 2016 Apr 14;6:24344. doi: 10.1038/srep24344 (PMC4830944; doi:10.1038/srep24344)
Supplement: Supplementary Information [file srep24344-s1.pdf]

Supplementary Document: Broadband photon pair generation in green fluorescent proteins through spontaneous four-wave mixing

Siyuan Shi[1,*], **Abu Thomas**[1], **Neil V. Corzo**[1], **Prem Kumar**[1], **Yuping Huang**[2,†], **and Kim Fook Lee**[1,‡]

[1]Center for Photonic Communication and Computing, Department of Electrical Engineering and Computer Science, Northwestern University, 2145 Sheridan Road, Evanston, IL 60208-3112, USA.
[2]Department of Physics and Engineering Physics, Stevens Institute of Technology, Hoboken, NJ, USA 07030
[*]siyuanshi2016@u.northwestern.edu
[†]Yuping.Huang@stevens.edu
[‡]kim.lee@northwestern.edu

ABSTRACT

This document provides supplementary material for ”Broadband photon pair generation in green fluorescent proteins through spontaneous four-wave mixing”.

Supplementary Information

Contents:

1. **The relative strength of dominant noise sources in organic fluorophores.**
2. **The signal and idler for the pyrromethene 556 sample.**
3. **The signal and idler for the pyrromethene 546 sample.**
4. **The signal and idler for the DCM in BzOH/EG and DCM in ethanol samples.**
5. **The signal and idler for the eGFP sample.**

sample	Non-Pol	Pol	$S_2^r;S_1^r(\uparrow;\downarrow;\|)$	noise source	CAR
DCM in BzOH/EG	$S_{2,s}^r$=0.94;$S_{1,s}^r$=0.06	$S_{2,s}^r$=0.89;$S_{1,s}^r$=0.11	$\downarrow;\uparrow$	FL+SE + SRS	$\approx$1
($P_p = 0.5\times10^{12}$)	$S_{2,i}^r$=0.64;$S_{1,i}^r$=0.36	$S_{2,i}^r$=0.58;$S_{1,i}^r$=0.42	$\downarrow;\uparrow$	FL+SE + SRS	
DCM in Ethanol	$S_{2,s}^r$=0.77;$S_{1,s}^r$=0.23	$S_{2,s}^r$=0.65;$S_{1,s}^r$=0.35	$\downarrow;\uparrow$	FL+SE + SRS	$\approx$1
($P_p = 0.5\times10^{12}$)	$S_{2,i}^r$=0.26;$S_{1,i}^r$=0.74	$S_{2,i}^r$=0.23;$S_{1,i}^r$=0.77	$\downarrow;\uparrow$	FL+SE + SRS	
Pyrromethene 556	$S_{2,s}^r$=0.87;$S_{1,s}^r$=0.13	$S_{2,s}^r$=0.88;$S_{1,s}^r$=0.12	$\|;\|$	FL+SE+SpRS	$\approx$1
($P_p = 2.5\times10^{12}$)	$S_{2,i}^r$=0.029;$S_{1,i}^r$=0.971	$S_{2,i}^r$=0.008;$S_{1,i}^r$=0.992	$\downarrow;\uparrow$	FL+SE + SRS	
Pyrromethene 546	$S_{2,s}^r$=0.30;$S_{1,s}^r$=0.70	$S_{2,s}^r$=0.46;$S_{1,s}^r$=0.54	$\uparrow;\downarrow$	SpRS	>1
($P_p = 2.5\times10^{12}$)	$S_{2,i}^r$=0.06;$S_{1,i}^r$=0.94	$S_{2,i}^r$=0.07;$S_{1,i}^r$=0.93	$\|;\|$	SRS	
eGFP	$S_{2,s}^r$=0.55;$S_{1,s}^r$=0.45	$S_{2,s}^r$=0.94;$S_{1,s}^r$=0.06	$\uparrow;\downarrow$	SpRS	>1
($P_p = 2.5\times10^{12}$)	$S_{2,i}^r$=0.22;$S_{1,i}^r$=0.78	$S_{2,i}^r$=0.20;$S_{1,i}^r$=0.80	$\|;\|$	SRS	

Table SI. The relative strength of dominant noise sources in organic fluorophores.

We can calculate $S_{2,s(i)}P_p^2/N_{s(i)}$ and $S_{1,s(i)}P_p/N_{s(i)}$ denoted as $S_{2,s(i)}^r$ and $S_{1,s(i)}^r$, respectively, at a chosen pump photon per pulse P_p for each sample as shown in Table SI. We can clearly see the relationships between the noise sources and the quantum correlation with CAR $\geq$1 by observing the increase ($\uparrow$), decrease ($\downarrow$) and unchanged ($\|$) of $S_{2,s(i)}^r$ and $S_{1,s(i)}^r$ with and without using the polarization filtering.

The eGFP and pyrromethene 546 share the similar feature after polarization filtering as follow; (i) the increase ($\uparrow$) of $S_{2,s}^r$ and the decrease ($\downarrow$) of $S_{1,s}^r$ for the signal where the dominant noise source is spontaneous Raman scattering, (ii) the unchanged of ($\|$) of $S_{2,i}^r$ and $S_{1,i}^r$ for the idler where the dominant noise source is the stimulated Raman scattering. Both DCM samples have the decrease ($\downarrow$) of $S_{2,s(i))}^r$ and the increase ($\uparrow$) of $S_{1,s(i)}^r$ for the signal and idler where their dominant noise sources are the fluorescence, stimulated emission, and stimulated Raman scattering processes.

There is a transition process of the noise sources in the signal and idler from the pyrromethene 546 $\rightarrow$ the pyrromethene 556 $\rightarrow$ the DCM in ethanol as follow; (i) the noise source of the signal has the transition from spontaneous Raman scattering in pyrromethene 546 $\rightarrow$ the fluorescence, stimulated emission, and spontaneous Raman scattering in pyrromethene 556 $\rightarrow$ the fluorescence, stimulated emission, and stimulated Raman scattering in the DCM in ethanol. (ii) the noise source of the idler has the transition from stimulated Raman scattering in pyrromethene 546 $\rightarrow$ the fluorescence, stimulated emission, and stimulated Raman scattering in pyrromethene 556 and the DCM in ethanol.

As shown in Fig. S1 for the DCM samples, the signal and idler are very much contaminated by the fluorescence and stimulated emission photons because the signal and idler show quadratic dependence on power. These noise sources are more dominant than spontaneous four-wave mixing process because of the CAR $\approx$1. Even though the efficiency of the stimulated four-wave mixing process for the DCM in BzOH/EG sample is high, the fluorescence, stimulated emission, and the stimulated Raman photons in the signal and idler prevent us from observing the quantum correlation of the signal and idler with CAR $>$ 1. We can select the wavelength of the signal and idler for avoiding the fluorescence and stimulated emission photons but not the stimulated Raman photons.

The spontaneous Raman photon, stimulated emission and the fluorescence photons are the main noise photons for the signal in the pyrromethene 556 sample as shown in Fig. S2. Even though the signal shows the quadratic dependence on power but the strengths of the fluorescence and stimulated emission are much stronger than spontaneous four-wave mixing process. The idler of pyrromethene 556 sample has more stimulated Raman photons than the fluorescence and stimulated emission photons because of the linear dependence on power as shown in Fig. S2. Even though the efficiency of the stimulated four-wave mixing process for the the pyrromethene 556 sample is higher than the eGFP, the noise photons bury the photon pairs generated through the spontaneous four-wave mixing process.

As for the pyrromethene 546 sample, the spontaneous Raman scattering is the main noise source for the signal but its quadratic dependence on power (Fig. S3) shows that the strength of spontaneous four-wave mixing process is stronger than the spontaneous Raman scattering. The idler has stimulated Raman photon because of its linear dependence on power. The light in the signal is less noisy and less contaminated by noise photons compared to the idler that resulting the maximum CAR of 6 and 15 for the scenario of without and with using polarization filtering, respectively.

The signal and idler generated in the eGFP sample show quadratic dependence on pump power (Fig. S4) as observed in the DCM samples. The light in the idler channel shows much clearer quadratic dependence on power compared to other samples. The maximum CAR of 45 and 145 for the scenario of without and with using polarization filtering indicate that the quadratic dependence on pump power is mainly due to the spontaneous four-wave mixing process. The signal shows stronger quadratic behavior than the idler because the stimulated Raman scattering process is stronger in the idler than spontaneous Raman scattering in the signal.

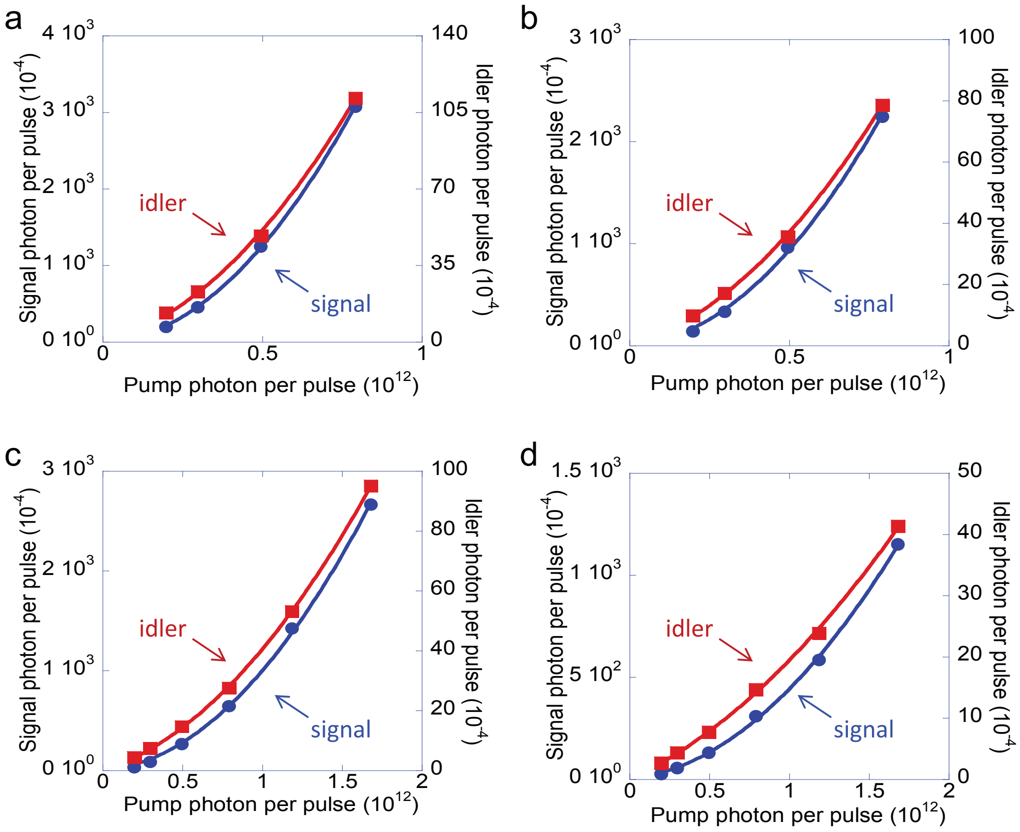

Figure S1. (Color online) The DCM in BzOH/EG sample, the signal and idler photon per pulse (a) without and (b) with using the polarization filtering. The DCM in ethanol sample, the signal and idler (c) without and (d) with using the polarization filtering.

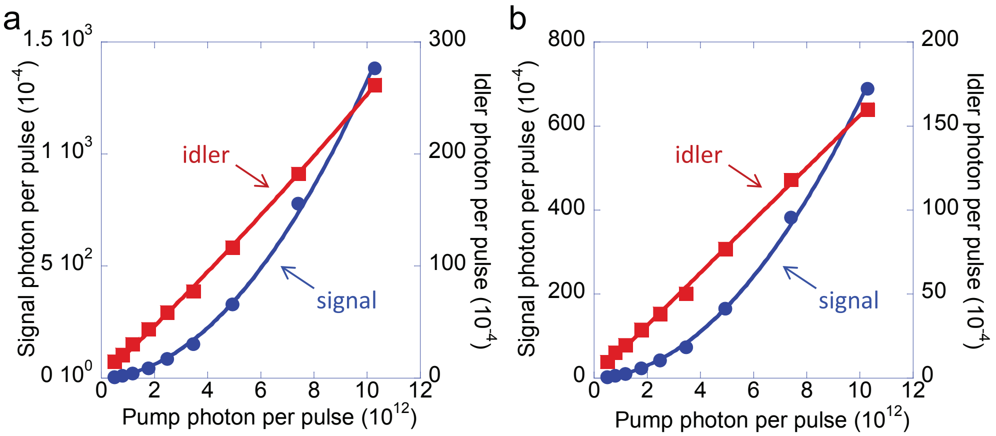

Figure S2. (Color online) The pyrromethene 556 sample, the signal and idler photon per pulse (a) without and (b) with using the polarization filtering.

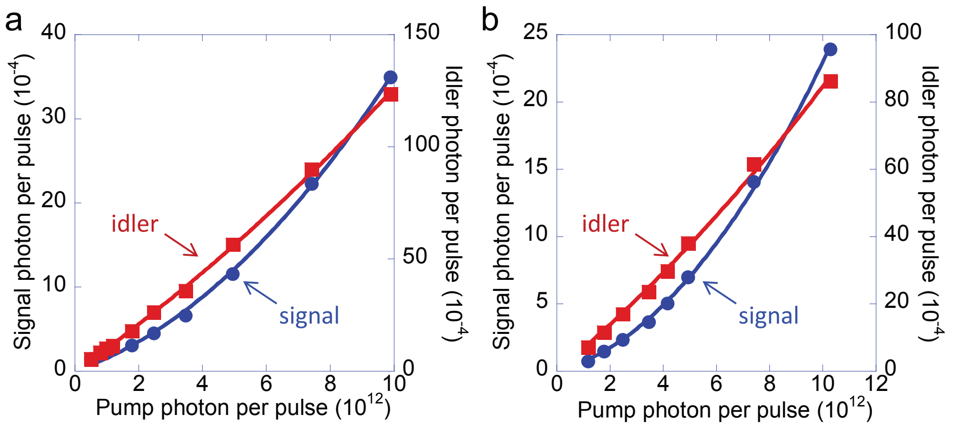

Figure S3. (Color online) The pyrromethene 546 sample, the signal and idler photon per pulse (a) without and (b) with using the polarization filtering.

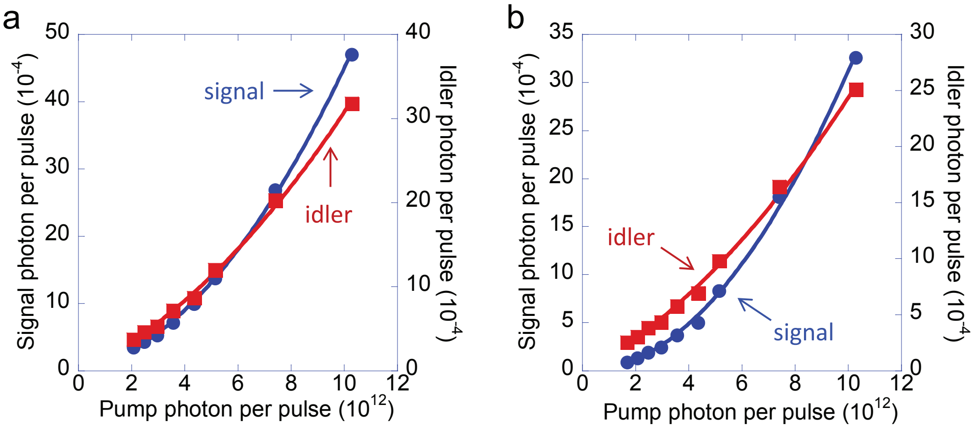

Figure S4. (Color online) The eGFP sample, the signal and idler photon per pulse (a) without and (b) with using the polarization filtering.
